# Trophoblasts, invasion, and microRNA

**DOI:** 10.3389/fgene.2013.00248

**Published:** 2013-11-21

**Authors:** Ludivine Doridot, Francisco Miralles, Sandrine Barbaux, Daniel Vaiman

**Affiliations:** Institut Cochin, INSERM U1016–CNRS UMR8104, Université Paris DescartesParis, France

**Keywords:** trophoblast, placental diseases, microRNAs, invasion mechanisms, angiogenesis, imprinted genes, oxygen, miR-210

## Abstract

MicroRNAs (miRNAs) have recently become essential actors in various fields of physiology and medicine, especially as easily accessible circulating biomarkers, or as modulators of cell differentiation. To this respect, terminal differentiation of trophoblasts (the characteristic cells of the placenta in Therian mammals) into syncytiotrophoblast, villous trophoblast, or extravillous trophoblast constitutes a good example of such a choice, where miRNAs have recently been shown to play an important role. The aim of this review is to provide a snapshot of what is known today in placentation mechanisms that are mediated by miRNA, under the angles of materno–fetal immune dialog regulation, trophoblast differentiation, and angiogenesis at the materno–fetal interface. Also, two aspects of regulation of these issues will be highlighted: the part played by oxygen concentration and the specific function of imprinted genes in the developing placenta.

## INTRODUCTION

**FIGURE 1:**
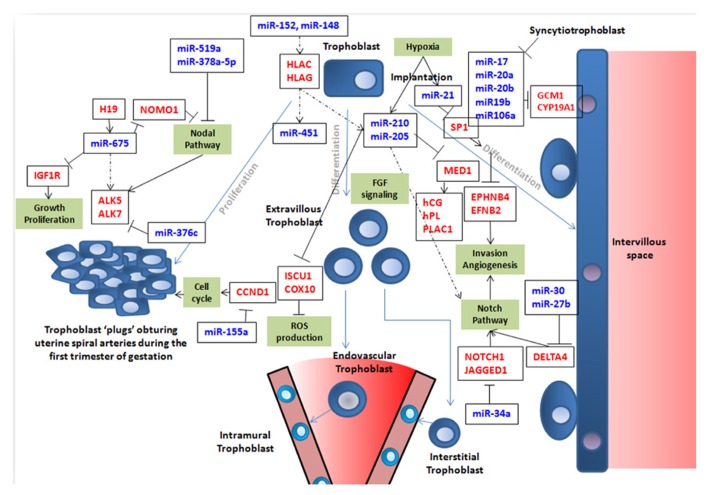
**This figure summarizes simplistically some of the features of trophoblast fate that are at least partly governed by miRNA expression.** Trophoblast cells are represented in blue with a round nucleus. Trophoblasts (in blue) can proliferate (left part of the figure) or differentiate into syncytiotrophoblast (right part of the figure; in this case, nuclei are filled in purple) or into endovascular or interstitial trophoblast in the vascular context of maternal arteries (represented as a cone in the lower part of the figure; in this case the nuclei are represented in light blue). In this latter case, they eventually become enclosed into the arterial wall to become intramural trophoblast along pathways that are still debated as nicely described by Pijnenborg et al. (2006). In the figure, crucial genes for cell cycle, Nodal, Notch, and FGF pathways, ROS production, invasion, and angiogenesis are presented in red. Crucial miRNAs are in blue. Blue arrows represent cell differentiation/proliferation pathways. Black arrows symbolize induction. When black lines are terminated by an orthogonal bar they represent inhibition. In the cases where the relation is not fully demonstrated, dotted arrows are used. It is clear that this vision is far from exhaustive, but attempts to depict current knowledge on the role of miRNA in building placental structure and ensuring efficient vascularization of this organ.

Mammalian pregnancy rests on a biological paradox: the intimate entangling of two different immunological systems during a long period of gestation (Medawar, 1961). In many species of mammals, the blastocyst enters the endometrial part of the uterus, during an invasive process occurring at the hatching of the blastocyst (4.5 days post coitum in mice, 7 days in humans). In humans, the external layer of the blastocyst (trophectoderm) proliferates, its outer cells syncytialize and lacunae form, defining digitations of the trophectoderm that form columns (primary chorionic villi). During the first trimester the existing structures persist and develop to form the floating villi of the placenta, while trophoblast cells that are not part of the villi form plugs sealing the intervillous space, and prevent maternal blood cells from being in contact with the syncytiotrophoblast (Carbillon et al., 2001). From the eighth and ongoing till at least the 16th week of gestation, the plugs begin to disappear, extravillous trophoblasts (EVTs) invade the uterine matrix, some going inside the decidua (interstitial trophoblasts), some fusing in a fashion similar to the syncytiotrophoblast (leading to placental bed giant cells), some replacing the maternal arteries endothelium in an ill-understood substitution process (endovascular trophoblasts), as depicted in **Figure 1**. The invasion of maternal tissue by trophoblast-derived cells is a specific and constant feature of human placentation (Goldman-Wohl and Yagel, 2002; Carter and Mess, 2007). Unsuccessful invasion is now recognized to lead to pregnancy diseases, such as preeclampsia (PE). PE is defined by a gestational hypertension accompanied by proteinuria that develops from the 20th week of gestation (Sibai, 2005). This disease has a prevalence estimated at ~3–7%, threatens the life of the mother and the fetus and its symptoms worsen from the appearance of the symptoms till the end of pregnancy. Delivery is the only real cure of this disease, which incidently is a major cause of iatrogenic prematurity. Genetic and epigenetic causes have been ascribed to PE (Chelbi and Vaiman, 2008).

The cells that compose these different structures derive from trophoblast stem cells, the fate of which has been particularly well-characterized in mice, and elegantly reviewed in Maltepe et al. (2010). This latter review emphasizes the involvement of epigenetic regulators in placental cell differentiation, such as DNA (cytosine-5)-methyltransferase 3-like (DNMT3L), various histone deacetylases (HDACs), suppressor of zeste 12 homolog (SUZ12). miRNAs are also important elements of the cell epigenetic machinery. Contrary to the other known players of epigenetic regulation, DNA methylation and post-translational histone modifications, they are able to affect gene expression after transcription by modulating mRNA stability and/or translation. The miRNAs achieve their epigenetic action by negatively modulating series of genes by targeting mRNA regulatory sequences often located in the 3’-untranslated sequence. Specifically, miRNAs are short (mean 22 nucleotides) ribonucleic acid (RNA) molecules. They are encoded by nuclear genes whose transcripts (called pri-miRNA) are processed in the nucleus to form pre-miRNAs through the action of the protein Drosha, or are part of introns that are also processed to form pre-miRNAs. Then, after export to the cytoplasm they are matured as miRNAs through the action of the protein Dicer through the RNA-induced silencing complex (RISC).

In the past 3 years, miRNAs have acquired a strong position in the list of factors influencing the different facets of trophoblast biology, such as proliferation, syncytialization, and invasion. A direct consequence, their impact on the major human placental diseases, such as PE, intra uterine growth restriction (IUGR, the failure of the fetus to reach a normal growth curve) or placenta accreta (a very severe obstetric disease where the placenta implants through the endometrium, and considerably increases the risk of hemorrhage at delivery) has also been revealed. A PubMed search for the keywords “miRNA” and “trophoblast” or “placenta,” yields 137 results, the first paper having been published in 2006. One seminal study that set the *de novo* landscape of miRNA-regulation in cells of the trophoblast lineage, was published in 2012 by Morales-Prieto et al. (2012). There, the authors screened 762 human miRNAs for their expression level in term and first trimester cytotrophoblasts, as well as in four cell lines: HTR8/SVneo (a cell line generated by transformation of an EVT), JEG-3 (a trophoblast-like line derived from a choriocarcinoma), ACH-3P and AC1-M59, the two latters consisting of choriocarcinoma fused either with early or late trophoblasts, respectively. One of the major outcomes of this work was the identification of clusters of placenta-specific miRNAs (C19MC, 54 miRNAs on chromosome 19, C14MC, 34 miRNAs on chromosome 14, and another minor cluster on chromosome 19). Their study also identified 27 miRNAs differentially expressed according to trophoblast age. Therefore this study gives an indicative encyclopedia of miRNAs susceptible to play an important role in the trophoblast.

In the present review, we will try to identify in the available literature the miRNAs that are known to be the major players of trophoblast function, in terms of (1) materno–fetal dialog leading to tolerance, (2) major differentiation events leading to syncytiotrophoblast generation from trophoblast cells, (3) angiogenesis and vasculogenesis in normal and pathological conditions, (4) impact of oxygen sensing, and (5) known links between miRNA and imprinted genes, since many of them are involved in placental function (Varrault et al., 2006; Renfree et al., 2013). We will mainly focus on those miRNAs for which validated gene targets have been identified. The orchestrator function of hypoxia in placental development will also be evoked. A limited number of miRNAs important for placental physiology are presented in **Figure 1** and some of them are summarized in **Table 1**. The classical mechanisms of miRNA production and maturation, through the action of Drosha or Dicer for instance, are beyond the scope of this review, although the description of the effects of Dicer inhibition on placental development will be mentioned. To obtain a clear vision of the mechanisms of miRNA maturation, the reader can consult the recent review from Chen and Wang (2013).

**Table 1:** Summary of some miRNAs discussed in the text and of their known function in placental physiology.

miRNA	Targets	Biological role	Reference
miR-152	HLA-G	Materno–fetal immune dialog	Tan et al. (2007), Kulkarni et al. (2011), Manaster et al. (2012)
miR-148	HLA-G, HLA-C	Syncytialization	Kumar et al. (2013)
miR-106a	hCYP191A1, hCGB, GCM1		
miR-19b	hCYP191A1, hCGB, GCM1		
miR-17, miR-20a, miR-20b	EPHB4, EFNB2	Spiral arteries remodeling, vasculogenesis, TGFβ signaling	Wang et al. (2012), Chen and Wang (2013)
miR-34a	Notch1, Jagged1		Pang et al. (2010)
miR-210	HOXA9, EPHA3		Pineles et al. (2007), Zhang et al. (2012)
miR-27b	Delta4, Spry2		Biyashev et al. (2012)
miR-126	SPRED1		Yan et al. (2013)
miR-29	MCL1, MMP2, VEGFA, ITGB1		Li et al. (2013b)
miR-378a-5p	Nodal		Luo et al. (2012)
miR-376c	ALK5/ALK7		Fu et al. (2013b)
miR-21		Oxygen sensing	Cindrova-Davies et al. (2013)
miR-548c			Ishibashi et al. (2012)
miR-205	MED1		Mouillet et al. (2010)
miR-424	FGFR1		Mouillet et al. (2013)
miR-21	SP1		Cindrova-Davies et al. (2013)
miR-210	ISCU1/2, COX10, HSD17B1		Pineles et al. (2007), Chen et al. (2010), Enquobahrie et al. (2011), Mayor-Lynn et al. (2011)
miR-518c	HSD17B1		Ishibashi et al. (2012)
cluster C19M	General pathways of development, differentiation and cell cycle	Imprinting	Flor et al. (2012)
miR-379	*DIO3*		
miR-431, miR-127, miR-136	*RTL1*		Girardot et al. (2012)
miR-675	Igf1R, NOMO1		Munir et al. (2004), Gao et al. (2012), Keniry et al. (2012)

## miRNAs AND REGULATION OF THE MATERNO–FETAL IMMUNE DIALOG

Implantation of the blastocyst occurs at 4.5 dpc in mice and at 7 dpc in humans. At this stage, the blastocyst establishes a physical contact with the endometrium during the implantation window. Hatching of the blastocyst allows the primary immunological contact between the fetal antigens and the maternal immune system. The conundrum of fetal non-rejection has been underlined by Peter Medawar as early as in the 1950s (Billington, 2003). One element of the solution is provided by the fetal expression of a limited antigen repertoire, since contrary to most cells of the human body, trophoblasts do not express human leukocyte antigen (HLA)-A and -B, the major histocompatibility (MHC) antigens that are highly polymorphic. Trophoblast cells express HLA-G (five alleles only; Hunt et al., 2006; one mRNA spliced in seven isoforms; Geraghty et al., 1987; Hunt et al., 2007) and HLA-C (two major alleles; Hiby et al., 2004; Moffett and Loke, 2006). Trophoblasts interact with uterine natural killer cells through their killer**-**cell immunoglobulin-like receptor (KIR) receptors (Hiby et al., 2010), two major forms of which exist (KIR-A and KIR-B). Some combinations of KIR and HLA-C have been shown to predispose to PE, but overall, the system is tuned to allow tolerance. To note, in recent years, HLA-G has been considered as a general immune-tolerogenic molecule in various tissues (Wiendl et al., 2005; Carosella, 2011; Gonzalez et al., 2012). HLA-G is expressed by EVT, and its expression has been found regulated by *miR-152* in the context of asthma (Tan et al., 2007). More recently, Manaster et al. (2012) showed that in EVT, HLA-G mRNA is targeted as well by *miR-148*, and this irrespectively to a specific single nucleotide polymorphism (SNP) located in the 3’-untranslated region (UTR) of HLA-G. This down-regulation is associated with an enhanced killing activity of natural killer (NK) cells. Very recently, it has been shown that in CD4^+^ T lymphocytes, soluble HLA-G modulates the expression of *miRNA-451* and *miRNA-210* (Morandi and Pistoia, 2013). These sets of results are extremely important in the context of human pregnancy. Among CD4^+^ T uterine lymphocytes, a small proportion (~5%) consists in Treg cells. These cells when maturated in peripheral organs, especially in uterine tissue, are key elements for a successful placentation in placental mammals (Samstein et al., 2012). HLA-C is also targeted by miRNAs, especially *miR-148* (Kulkarni et al., 2011) although the impact of this regulation has mainly been studied in the context of HIV infection with the description of a sensibility-modulating SNP in the 3’-UTR of the gene. This regulation of HLA-C by miRNA has not been studied in the trophoblast context, nevertheless it is strongly expressed in cells of trophoblast origin (Manaster et al., 2012), suggesting that it may play a role in the expression of this antigen. To summarize, miRNAs appear as able to modulate very early the immunological balance of the uterine–embryo contact.

## miRNAs AND THE TROPHOBLAST–SYNCYTIOTROPHOBLAST TRANSITION

Besides these very early effects on trophoblast biology, miRNAs have recently been shown to play extensive roles on the fate of trophoblasts. One of them is the evolution into syncytiotrophoblast. In fact near the end of pregnancy, villous trophoblasts become very rare, limiting the rejuvenation capability of the syncytiotrophoblast (Rigourd et al., 2009). Recently, Kumar et al. (2013) interrogated by microarray analysis the developmental transition leading to syncytiotrophoblast differentiation. In this study, the authors identified miRNAs belonging to three clusters modified during trophoblast differentiation (*miR-17, 20a, 20b, 19b* belonging to the miR-17~92 cluster, on chromosome 13q31; *miR-106a*, on chromosome X, belonging to cluster 106a~363, and *miR-93*, on chromosome 7, belonging to the miR 106b~25 cluster). These miRNAs are all down-regulated in syncytiotrophoblast. *miR-106a* and *miR-19b* directly target the 3’-UTR of the aromatase (*hCYP19A1*) gene. Aromatase is expressed in the syncytiotrophoblast exclusively. One of the most interesting targets discovered by the authors is glial cell missing homolog1 (*GCM1*), a transcription factor playing a major function in trophoblast differentiation and fusion (Anson-Cartwright et al., 2000), also known to be down-regulated in PE, one of the most important diseases of pregnancy (Chen et al., 2004; Chiang et al., 2009; Drewlo et al., 2011; Wilson et al., 2011). *GCM1 *expression is also reduced fourfold in JEG-3 cells overexpressing STOX1 that share strong transcriptomic characteristics with the human preeclamptic placenta (Rigourd et al., 2008, 2009). Interestingly, one of the most classical markers of syncytialization, human chorionic gonadotrophin (hCGß), was also decreased by miR-19b and miR106a; in the absence of predicted *bona fide* target in the gene, it was shown that this results from an indirect action of GCM1 (Kumar et al., 2013). In the same study the authors show that c-Myc binds directly to the 5’ upstream region of the clusters miR-17~92 and miR-106a~363, the expression of this protooncogene being decreased during syncytiotrophoblast differentiation. These novel mechanistic data especially on the miR-17~~92 and miR-106a~363 clusters, strengthen recent observations published by Wang et al. (2012) that show up-regulation of *miR-17*, *miR-20a* (belonging to miR-17~92) and *miR-20b* (belonging to miR-106a~363) in preeclamptic placentas, which as mentioned in the introduction is a disease generally caused by trophoblast dysfunction and presumably associated to a generalized activation of the endothelium.

## miRNAs, ANGIOGENESIS, AND VASCULOGENESIS AT THE UTERO-PLACENTAL INTERFACE: REGULATION OF THE NOTCH PATHWAY AND LINKS WITH PREECLAMPSIA

A key event in early placentation involves the remodeling of the uterine spiral arteries by migrating EVTs a process known as pseudovasculogenesis or vascular mimicry (Ji et al., 2013). This process transforms the uterine spiral arteries from high resistance, low flow muscular vessels to low resistance, and high flow vessels enabling them to ensure proper irrigation to the developing placenta/fetus. It has been consistently reported that in early onset PEs (generally the most severe) the remodeling of the spiral arteries is impaired (Red-Horse et al., 2004). This leads to reduced perfusion of the developing placenta, subsequent intermittent hypoxia-reoxygenation episodes, reactive oxygen (ROS) production, cell injury, inflammation, and liberation of placental factors and debris into the maternal circulation. Spiral artery remodeling appears to be a multi-stage process involving: spiral arteries preliminary remodeling (characterized by vacuolization and apoptosis of resident ECs), EVTs migration and invasion of the decidua and myometrium, incorporation of EVTs into the spiral arteries vessel walls, and acquisition by the endovascular EVTs of an EC-like phenotype. The trans-differentiation of EVTs into EC-like cells (vascular mimicry) results in the down-regulation of epithelial cell-type markers E-cadherin and integrin α6β4 and the induction of the expression of endothelial adhesion molecules such VE-cadherin, PECAM, NCAM as well as integrins α5β1, α1β1, αVβ3 (Damsky and Fisher, 1998).

Experimental evidence of the implication of miRNAs in the different stages of spiral arteries remodeling and their eventual dysfunction in PE is still limited. A recently published study reports an increased expression of members of the miR-17 family miRNAs (*miR-17*, *miR-20a*, and *miR-20b*) in PE versus normotensive human placentas (Wang et al., 2012). Among the potential targets of the miR-17 family miRNAs the authors identified the ephrin receptor B4 (*EPHB4*) and ephrin B2 (*EFNB2*), two Eph receptor tyrosine kinases involved in angiogenesis (Wang et al., 1998). In the placenta, the couple EFNB2/EPHB4 directs EVTs invasion away from the placenta and towards uterine spiral arterioles (Red-Horse et al., 2005). The same authors found that the forced expression of *miR-20a* in BeWo cells (a human cell line derived from a placental choriocarcinoma) inhibits syncytialization and induces the expression of ECs markers. Therefore, it has been postulated that in normal placentation low levels of miR-17 family miRNAs allow a higher expression of EFNB2/EPHB4 in the EVTs facilitating the invasion and uterine spiral arteries remodeling. Ephrin receptors B2 and B4 are receptor tyrosine kinases and several reports suggest their involvement in cell differentiation mechanisms (Adams, 2002). They are also involved in vascular endothelial growth factor (VEGF)-mediated angiogenic functions through regulation of signaling of the VEGFR2 and VEGFR3 receptors in endothelial cells (Sawamiphak et al., 2010; Wang et al., 2010). In PE, an increased expression of miR-17 family members would suppress the expression of *EFNB2*/*EPHB4* resulting in inhibition of spiral arteries invasion and endovascular EVTs differentiation (Chen and Wang, 2013).

Recent studies have implicated the Notch pathway in the process of spiral arteries remodeling and in the pathogenesis of PE (Hunkapiller et al., 2011). This is in good accordance with the known role of Notch signaling in vascular patterning and in the specification of arterial identity (Roca and Adams, 2007; Swift and Weinstein, 2009). The core component of this pathway consists of four transmembrane receptors (Notch1–4) and five ligands (Delta1/3/4 and Jagged 1/2) that are dynamically expressed through placental development (Hunkapiller et al., 2011). Conditional deletion of *Notch2* in mice leads to significant reduction in placental perfusion and arterial invasion by trophoblasts (Gasperowicz and Otto, 2008). Also, when human cytotrophoblasts are grown on Matrigel, NOTCH promotes their invasion and acquisition of an arterial EC-like phenotype marked by EFNB2 up-regulation (Gasperowicz and Otto, 2008). Moreover, significant *JAGGED1* down-regulation in the perivascular and endovascular EVTs has been detected in PE, and found to be associated with failed vascular remodeling (Gasperowicz and Otto, 2008). Interestingly, overexpression of *miR-34a* in the choriocarcinoma cell line JAR (derived from a trophoblastic tumor of the placenta) inhibits cell invasion by targeting *Notch1* and *Jagged1* (Pang et al., 2010). This suggests a possible role of miRNAs in the regulation of Notch signaling in the early stages of uterine invasion and spiral arteries remodeling. Recently, we have shown that while pri-miR-34a is overexpressed twice in preeclamptic placentas (where the gene promoter is demethylated), the mature miR-34a is present at a much lower level in pathological placentas than in normal placentas (Doridot et al., 2013). Our data suggest that this mechanism could contribute to an indirect up-regulation of SERPINA3 in pathological pregnancies (Chelbi et al., 2007, 2012), this gene being an important actor of placental homeostasis and trophoblast invasion.

Many other miRNAs are known to be involved in the regulation of Notch signaling. Among these, *miR-30* targets *DLL4* (delta like ligand-4; Bridge et al., 2012), and *miR-210* over-expression in ECs causes up-regulation of Notch1 and induces migration and tube formation in Matrigel (Lou et al., 2012). Interestingly, miR-210 is known to be one of the most up-regulated miRNAs in the preeclamptic placenta and has been involved in trophoblast migration and invasion process (Pineles et al., 2007; Zhang et al., 2012). Another recent study has shown that *miR-27b* controls two critical vascular functions in zebrafish: it turns on the angiogenic switch by promoting endothelial tip cell fate and sprouting and it promotes venous differentiation. The Notch ligand Delta4 and Sprouty homolog 2 (Spry2) were identified as miR-27b targets involved in these functions. Moreover, miR-27b is necessary for the formation of the first embryonic vein in fish and controls the expression of arterial and venous markers in human endothelium, including EFNB2, EPHB4, fms-related tyrosine kinase 1 (Flt1), and fms-related tyrosine kinase 4 (Flt4; Biyashev et al., 2012). Also, in vascular smooth muscle cells (VSMCs) Notch signaling through Jagged1 promotes a differentiated phenotype characterized by an increased expression of contractile proteins, this effect being mediated through the induction of *miR-143/145* transcription (Boucher et al., 2011). Finally, several miRNAs (miR-9, miR-21, miR-27, miR-124, miR-130, miR-148, and miR-181) involved in vascular development and ECs signaling have been predicted to target components of the Notch pathway (Anand, 2013). Therefore, miRNAs regulation of Notch signaling might play an important role in placental angiogenesis.

In a larger context, the importance of miRNAs in angiogenesis and ECs physiology has been established by the study of Dicer inactivation. Thus, Dicer hypomorphic mice present severely impaired vascular development in both the embryo and yolk sac (Yang et al., 2005). Endothelial specific deletion of Dicer in mice decreases postnatal angiogenesis (Suarez et al., 2008). Also, VSMCs inducible deletion of Dicer in mice, leads to impaired differentiation, contractility, and vascular remodeling (Albinsson et al., 2011). Altogether, these studies point out to the essential role of Dicer and hence of miRNAs for the proper development and function of vascular cells. The analysis of the miRNAs expressed in HUVECs has allowed identifying miRNAs involved in ECs function (Poliseno et al., 2006). Among these *miR-126* has been found as one of the most abundantly expressed in ECs (Fish et al., 2008; Wang et al., 2008). This miRNA is essential for angiogenesis and targets Sprouty related EVH domain containing protein 1 (SPRED1), a negative regulator of vascular endothelial growth factor 1 (VEGF). Owing to its role in endothelial progenitor cells (EPCs) physiology, a recent study has suggested that miR-126 is essential for placental vasculogenesis and suggested that it could provide a therapeutic approach for PE (Yan et al., 2013). Another study has shown that *miR-29* induces apoptosis and inhibits the invasive and angiogenic capacities of trophoblast cell lines (HTR-8/SVneo, BeWo, and JAR). This study has shown that miR-29b regulates the expression of myeloid cell leukemia sequence 1 (MCL1), matrix metalloproteinase 2 (MMP2), vascular endothelial growth factor A (VEGFA), and integrin β1 (ITGB1) genes by directly binding their 3’-UTRs. Moreover, an inverse correlation was detected between miR-29b and the expression levels of these targets genes in PE (Li et al., 2013b).

In conclusion, to date many miRNAs have been found to play important roles in the regulation of angiogenesis and ECs homeostasis (Hartmann and Thum, 2011; Small and Olson, 2011), an issue indirectly linked to trophoblast differentiation. Indeed, oxygen availability or scarceness are landmark morphogens for trophoblast cell fate.

Despite an increasing number of studies seeking to identify miRNAs involved in the pathogenesis of PE, only a few of them have been associated to the essential process of placental angiogenesis, and in particular to its earliest steps (Fu et al., 2013a). In humans, the analysis of the role of miRNAs in spiral arteries remodeling is complicated due to the difficulty to access to placental tissue for experimental purposes. Therefore, it is likely that further studies will be greatly dependent on animal models (i.e., mice), but also, owing to the particularities of human placentation, on the development of *in vitr*o models able to reproduce the different stages and cellular interactions (cell contacts, cytokines signaling, miRNA exchanges mediated by exosomes, etc.) which are likely involved in spiral artery transformation.

## miRNAs AND OXYGEN SENSING AT THE TROPHOBLAST LEVEL

Hypoxia is a key signal for normal placental development in physiological pregnancy, and has a crucial role in the control of trophoblast differentiation into invasive or proliferating cells (Zhou et al., 1997; Jauniaux et al., 2003). Heretofore, its effect was primarily described as mediated through HIF transcription factors, but recently, it was recognized that miRNAs induced by hypoxia could participate by regulating genes that are not directly hypoxia-inducible (Huang et al., 2009). Hypoxia-inducible miRNAs constitute a new group of genes that could be important for trophoblast differentiation. To identify such miRNAs, trophoblast cells purified from term placentas were grown in normoxia or hypoxia (48 h, 1% O_2_), and miRNAs expression profile was studied by microarray and Northern blot (Mouillet et al., 2010). Among the most highly expressed miRNAs, seven miRNAs were shown modified in hypoxia: *miR-93*, *miR-205*, *miR-224*, *miR-335*, *miR-424*, *miR-451*, and *miR-491*. Other miRNAs induced by hypoxia were identified by targeted approaches, as they were shown to be modified in preeclamptic placentas (considered as hypoxic) or pregnancies associated with abnormal placental Doppler waveforms, indicative of a blood circulation defect; this was the case for *miR-21* (Cindrova-Davies et al., 2013), *miR-210* (Ishibashi et al., 2012; Lee et al., 2012; Muralimanoharan et al., 2012), and *miR-518c* (Ishibashi et al., 2012). These hypoxia-induced modifications in miRNA content are probably not linked to miRNAs biogenesis, since the responsible enzymes are apparently not modified by hypoxia (Donker et al., 2007).

For some of these candidate miRNAs, their potential involvement in trophoblast function and/or differentiation was assessed thanks to the identification of their target genes. *miR-205* concentration is increased in hypoxic conditions (around twice after 48 h at <1% O_2_) in primary trophoblast cells and miR-205 targets *MED1*, as validated by luciferase assay (Mouillet et al., 2010). MED1 is an important regulator of murine placental development (Landles et al., 2003), that regulates the expression of hCG, human placental lactogen (hPL), and placental-specific protein 1 (Plac1) in primary trophoblast cells (Mouillet et al., 2010). *miR-424* is down-regulated around fivefold following 72 h of hypoxia in primary trophoblast cells (but not in the trophoblast cell line JEG-3). It targets FGFR1 as demonstrated by luciferase assay (Mouillet et al., 2013). FGF signaling has been implicated throughout development in many signaling pathways controlling cellular proliferation, differentiation, survival, and angiogenesis (Turner et al., 2010). FGFR1 role in pregnancy is however unclear, since its deletion in mice is embryonically lethal around E7.5–E9.5, preventing a detailed analysis of its role in the placenta (Deng et al., 1994; Yamaguchi et al., 1994). miR-424 amounts are also restricted by other signals inhibiting differentiation (in syncytiotrophoblast cells), such as dimethyl sulfoxide (Mouillet et al., 2013). In the same article, *miR-503*, localized near miR-424 on chromosome X, was shown to exhibit the same expression profile in primary trophoblast cells, suggesting the existence of a polycistronic precursor containing both miRNAs. *miR-21* is elevated in placentas with abnormal Doppler waveforms (IUGR or preeclamptic) compared to control or preeclamptic placentas with normal Doppler waveforms (around twice; Cindrova-Davies et al., 2013). Furthermore, in placental explant submitted to 3 h of hypoxia (0.5% O_2_) followed by 20 h at 10% O_2_ in order to mimic hypoxia/reoxygenation injury, there is an up-regulation of miR-21 (1.6-fold). This miRNA was shown to directly target SP1, which in turn controls the expression of cystathionine-γ-lyase (CSE), responsible for hydrogen sulfide (H_2_S) synthesis (Yang et al., 2012). Yet H_2_S is thought to have a vasodilator effect, which could be important for a correct placentation. This is substantiated by the decreased expression of CSE in placentas with abnormal Doppler waveforms, and in the placental explant submitted to hypoxia/reoxygenation injury (Wang et al., 2013b). To note, SP1 a zinc finger transcription factor is recurrently found important in placental physiology modulation (Gascoin-Lachambre et al., 2010; Chelbi et al., 2012; Vaiman et al., 2013). miR-21 could have other effects on trophoblast function in terms of proliferation, since it has been described as a pro-invasive oncogene in breast cancer (Xu et al., 2008). *miR-210* is probably the miRNA for which there is the most abundant literature in relation with placental hypoxia. Actually, it was one of the first identified hypoxia-up-regulated miRNAs, principally in the context of tumors (Kulshreshtha et al., 2007). Since then, miR-210 was shown to be induced by hypoxia in a vast number of cell types (Hsu et al., 2012), leading to rename it “hypoxamiR.” miR-210 was also found affected in every miRNA screen comparing preeclamptic and normal placentas (Pineles et al., 2007; Santner-Nanan et al., 2009; Enquobahrie et al., 2011; Mayor-Lynn et al., 2011). miR-210 targets notably iron sulfur cluster scaffold proteins (ISCU1/2; Chan et al., 2009; Chen et al., 2010) and cytochrome c oxidase assembly protein (COX10; Chen et al., 2010). ISCU1/2 facilitate the assembly of iron-sulfur clusters, prosthetic groups that are critical for electron transport and mitochondrial oxidation–reduction reactions, while COX10 is essential for the assembly of electron transport system complexes I and IV showing how miR-210 may strongly impact mitochondrial function. Colleoni et al. (2013) confirmed recently that such mitochondrial alterations occur in trophoblast cells by cultivating JEG-3 cells for 4 days in hypoxia (1% O_2_) and using high altitude placentas, in which miR-210 is around twice higher than in sea-level placentas, demonstrating a diminished protein level for complexes I and IV. Mitochondrial function following miR-210 induction by hypoxia was also assessed in preeclamptic placentas and primary trophoblast cells isolated from control placentas (Muralimanoharan et al., 2012). In this latter study, the authors demonstrated mitochondrial dysfunction in placentas from pregnancies complicated by PE, associated with elevated ROS production and HIF-1α stabilization, up-regulation of miR-210 and down-regulation of ISCU. They demonstrated that increased miR-210 is necessary and sufficient for explaining the observed mitochondrial dysfunction, by using gain of function and loss of function approaches. miR-210 effect on iron metabolism could also impact the invasive capacity of trophoblast as suggested by a decreased invasion of human first-trimester trophoblast cells in Matrigel^TM^ following ISCU knock-down (Lee et al., 2011). However the invasive defect following miR-210 expression can also be due to other validated targets such as EphrinA3 and HOXA9, both involved in migration and vascular remodeling (Zhang et al., 2012). In this latter study, the authors also demonstrated that miR-210 expression is also regulated by NF-κB (p50 subunit), in addition to HIF1α. MiR-210 also targets hydroxysteroid (17-β) dehydrogenase 1 (HSD17B1), a steroidogenic enzyme gene predominantly expressed in the placenta, the encoded protein catalyzing the conversion of estrone to 17-β estradiol (Ishibashi et al., 2012). This gene is also the target of *miR-518c*, another miRNA expressed in the syncytiotrophoblast layer of the placenta. These two miRNAs were found by comparing miRNA profile from normal and preeclamptic placentas, by high throughput sequencing and qPCR. Luciferase assays confirmed HSD17B1 as a genuine target of miR-210. miR-518c and miR-210 were then assessed in JEG-3 and BeWo cells exposed to hypoxia (1% O_2_) and demonstrated to be induced. This study led to propose to use HSD17B1 as a biomarker, as it was shown reduced in the plasma from preeclamptic patients, before the onset of the symptoms. However, how the reduced HSD17B1 could participate to the physiopathology remains unclear.

For most identified hypoxia-up-regulated miRNAs in trophoblast cells, there are neither identified specific target genes, nor explanations about a possible involvement in trophoblast cell function. However, description of some targets found in other cell type can lead to interesting hypotheses. For example, overexpression of *miR-93* attenuated hypoxia-induced apoptosis in endothelial and skeletal muscle cells, enhanced proliferation in both cell types, and enhanced endothelial cell tube formation (Hazarika et al., 2013). *miR-224* was recently shown to target Raf kinase inhibitor protein (RKIP), a tumor suppressor that protects against metastasis and genomic instability, and to have a positive effect on Transwell migration, 3D growth in Matrigel, and wound healing in breast cancer cell lines, probably through RKIP target genes, notably MMP1 (Huang et al., 2012). *miR-335* overexpression can simultaneously suppress the invasiveness of ovarian cancer cells and lung cancer cells and promote apoptosis of lung cancer cells by targeting Bcl-w (Cao et al., 2013; Wang et al., 2013a). *miR-451* inhibits cell proliferation in human hepatocellular carcinoma through direct suppression of IKK-β (via the down-regulation of cyclin D1 and c-Myc; Li et al., 2013a). Finally, *miR-491* is involved in the migratory capacity of hepatocellular carcinoma cells by inhibition of matrix metalloproteinases (Yu et al., 2013). In conclusion, all these recent studies involve hypoxia-up-regulated trophoblast miRNAs in the control of proliferation, migration, or invasion, all key processes in trophoblast function. Given the pivotal role of oxygen, playing on this factor on purified trophoblast, before or after syncytialization could help characterizing exhaustively the miRNAs involved and shed a novel light on the pathways of trophoblast differentiation in physiology and pathology.

## miRNAs AND REGULATION OF IMPRINTED GENES IN TROPHOBLAST AND PLACENTAL BIOLOGY

The chromosome 19 miRNA cluster that we mentioned in the introduction (C19MC) is hosted in a 100-kb region located on human chromosome 19q13 and contains 46 tandemly repeated miRNAs (Bortolin-Cavaille et al., 2009). The cluster is submitted to parental imprinting, resulting in the expression of the primary long transcript exclusively from the paternal copy, from which introns containing the different miRNAs are spliced (Bortolin-Cavaille et al., 2009; Noguer-Dance et al., 2010). This regulation is under the control of a CpG rich region that undergoes an extensive methylation only in the maternal gametogenesis, resulting in the establishment of a differentially methylated region (DMR) upstream the cluster. Other imprinted genes are located in the neighborhood of this cluster, including *PEG3* and *ZNF331*. These C19MC miRNAs are specific of the primate lineage and are expressed almost exclusively in embryonic stem cells and the placenta (Noguer-Dance et al., 2010).

Expression of the C19MC miRNAs can be detected in trophoblastic cells but also in stromal cells where *miR-520c-3p*, *miR519a-3p*, and *miR517a-3p* are expressed, whatever the gestational age (Flor et al., 2012). Trophoblastic model cell lines such as JEG and BeWo seem to express them whereas HTR8 cells mimicking EVTs do not. Some differences can also be observed depending on the differentiation stage of the cells. The presence of these miRNAs could also be observed within exosomes, vesicles that are secreted by the syncytiotrophoblast layer, released in the feto–maternal circulation and that are likely to participate to exchanges between the fetus via its placenta, and the mother (Donker et al., 2012). An abnormal expression of these miRNAs is observed in some cancers, suggesting an oncogenic function and giving clues concerning their possible physiological function. For example the overexpression of a C19MC miRNA, *miR-519d*, in hepatic cancers is associated with an increased proliferation and invasion, by direct targeting of molecules regulating the cell cycle such as *p21*, *PTEN*, *AKT3*, and *TIMP2* (Fornari et al., 2012). In the developing placenta, C19MC miRNAs could participate to the trophoblastic proliferation necessary to constitute a large placenta required in primates and to the deep invasion of the maternal decidua necessary to establish a correct blood flow to fulfill fetal needs. If direct interactions between the C19MC miRNAs and proved targets have not been extensively described, predicted targets indeed belong to general pathways of development, differentiation and cell cycle. Within the cluster, seed sequences are mostly shared by the different genes (Lin et al., 2010). miR520c-3p seems to be the only one of the cluster responding positively to hypoxia.

Located within a large imprinted domain on chromosome 14q32, the C14MC cluster contains 53 miRNAs tandemly repeated in two segments overlapping about 40 kb. The region also shelters the *MEG3*, *DLK1*, *RTL1*, and *DIO3* imprinted coding genes (Morales-Prieto et al., 2012). The C14MC miRNAs are expressed only from the maternal allele as *MEG3* whereas their neighbor coding genes *DIO3*, *RTL1*, and *DLK1* are paternally expressed, all under the control of a specific upstream DMR. This profile is conserved in the mouse genome and the cluster appears to be eutherian-specific. Some C14MC miRNAs directly target the nearby imprinted genes *DIO3* (*miR-379*), *RTL1* (*miR-431*, *miR-127*, *miR-136*), suggesting a role in placental development (Girardot et al., 2012). Indeed, mice null for Rtl1 or overexpressing it display an abnormal development and anomalies of the placenta, notably in capillary formation (Sekita et al., 2008). Their expression seems to be concentrated in the developing embryo and placenta. Their predicted targets and expression profiles could propose a role in stem cell physiology and pluripotency.

The IGF2-H19 locus is a well-deciphered imprinted locus located on human chromosome 11p15 and murine chromosome 7. While the *IGF2* gene codes for the insulin-like growth factor 2, *H19* is a large non-coding RNA. Both genes are epigenetically regulated under the control of a main imprinting control region (ICR) and in opposite ways: *IGF2* is expressed from the paternal allele while *H19* is maternally expressed. If the function of IGF2 as a fetal and placental growth-promoting factor is well known, the mechanisms of action of *H19* remain less clear. A tumor-suppressive effect has been observed in mice in a model of colorectal cancer (Yoshimizu et al., 2008). In particular, 2 miRNAs *miR-675-5p* and *miR-675-3p*, are produced from the first exon of H19, the region showing the highest conservation within mammals. Though H19 is widely expressed in various fetal tissues, miR-675 is only abundant and increasingly expressed in placental tissues, in the labyrinthe from E11,5. The maturation of miR-675 seems to be blocked in the first part of gestation by the binding of the HuR protein (Keniry et al., 2012). There is an inverse correlation between the decrease of HuR and the increase of miR-675 expression, together with the slowing or arrest of placental growth during gestation time. In particular, Igf1R, the main receptor of Igf2, is a target of miR-675. The authors hypothesized that miR-675 plays a role in controlling placental growth and size along gestation and that H19 serves as a reservoir of miR-675, produced in response to stress or particular needs during gestation (Keniry et al., 2012). Nodal modulator1 (NOMO1) is a direct demonstrated target of miR-675 (Gao et al., 2012). Down-regulation of H19/miR-675 and increased expression of NOMO1 are observed in preeclamptic placentas. NOMO1 participates to the Nodal signaling pathway involved in trophoblast proliferation control and apoptosis (Munir et al., 2004). H19 inhibition in JAR cells resulted in the abolition of apoptosis and proliferation (Yu et al., 2013). Besides the involvement of imprinted genes in Nodal modulation it is worth mentioning the important recent findings of Luo and co-workers, that after identifying a potential binding site for *miR-378a-5p* in the 3’ UTR of Nodal, demonstrated this targeting. Functionally speaking, the authors showed that overexpression of the pre-miR (miR-378) induces several enhancements of trophoblast function in HTR8 cells (cell survival, proliferation, migration, invasion) and outgrowth and spreading of EVT from first trimester placental explants (Luo et al., 2012). Similarly, the same team showed that *miR-376c* targets ALK5 and ALK7 (receptors for transforming growth factor-β and Nodal, respectively), its overexpression leading to improved trophoblast “behavior” as that of miR-378a-5p (Fu et al., 2013b). It is interesting to notice that miR-378a-5p is reduced in PE. Overall, these results emphasize the role of Nodal signaling in trophoblast function.

## CONCLUSIONS

The number of articles published on miRNA and trophoblast development has been steadily increasing every year since 2006, to reach 37 in 2012, and 33 in July 2013 when this review has been written. This increase clearly illustrates the impact of miRNAs in placental physiology and pathology. The present review is clearly not exhaustive, mainly because of space restriction. It nevertheless gives an overview of several of the major aspects where miRNAs play a physiological role in placental development and trophoblast differentiation. In this context, the links between imprinted genes, oxygen signaling, proliferation, angiogenesis, and invasion in connection with miRNA expression will likely be better understood in the future.

## Conflict of Interest Statement

The authors declare that the research was conducted in the absence of any commercial or financial relationships that could be construed as a potential conflict of interest.
